# Clinical validity assessment of genes for inclusion in multi‐gene panel testing: A systematic approach

**DOI:** 10.1002/mgg3.630

**Published:** 2019-03-21

**Authors:** Tricia N. Zion, Bess Wayburn, Sourat Darabi, Devon Lamb Thrush, Erica D. Smith, Tami Johnston, Brissa Martin, Kelly D. F. Hagman, Melissa Parra, Christian Antolik

**Affiliations:** ^1^ Ambry Genetics Aliso Viejo California

**Keywords:** cardiology, clinical validity, detection rate, gene characterization, gene vetting, gene–disease relationship, multi‐gene panel genetic testing

## Abstract

**Background:**

Advances in sequencing technology have led to expanded use of multi‐gene panel tests (MGPTs) for clinical diagnostics. Well‐designed MGPTs must balance increased detection of clinically significant findings while mitigating the increase in variants of uncertain significance (VUS). To maximize clinical utililty, design of such panels should include comprehensive gene vetting using a standardized clinical validity (CV) scoring system.

**Methods:**

To assess the impact of CV‐based gene vetting on MGPT results, data from MGPTs for cardiovascular indications were retrospectively analyzed. Using our CV scoring system, genes were categorized as having definitive, strong, moderate, or limited evidence. The rates of reported pathogenic or likely pathogenic variants and VUS were then determined for each CV category.

**Results:**

Of 106 total genes, 42% had definitive, 17% had strong, 29% had moderate, and 12% had limited CV. The detection rate of variants classified as pathogenic or likely pathogenic was higher for genes with greater CV, while the VUS rate showed an inverse relationship with CV score. No pathogenic or likely pathogenic findings were observed in genes with a limited CV.

**Conclusion:**

These results demonstrate the importance of a standardized, evidence‐based vetting process to establish CV for genes on MGPTs. Using our proposed system may help to increase the detection rate while mitigating higher VUS rates.

## 1 INTRODUCTION

The human genome contains approximately 19,000 genes, of which approximately 4,000 are reported in the Online Mendelian Inheritance in Man (OMIM) database to be associated with Mendelian phenotypes (Boycott et al., 2017; Chong et al., 2015; Ezkurdia et al., 2014; Smith et al., 2017). As knowledge of genetic and phenotypic diversity expands along with advances in sequencing technology, the use of multi‐gene panel tests (MGPTs) in clinical diagnostics has increased significantly. As novel genomic variants continue to be identified in genes previously not known to be associated with disease, the clinical interpretation of this variation constitutes a great challenge to genomic medicine. With continual increase in the number of genes reported to be associated with disease, the assessment of the strength of gene–disease relationships, also referred to as clinical validity (CV), will be crucial in the interpretation of genomic variants reported through clinical molecular diagnostics (Ghouse et al., 2017).

MGPTs with many genes may have a higher likelihood of detecting clinically significant findings, but may also increase the number of variants of uncertain significance (VUS) identified (Ouellette et al., 2017; Pugh et al., 2014). According to the American College of Medical Genetics (ACMG) variant classification guidelines, pathogenic and likely pathogenic variants are clinically actionable while VUS are not (Richards et al., 2015). Therefore, MGPTs offered by clinical diagnostic laboratories must balance increased detection rates of clinically actionable findings while mitigating the increase in VUS rate as much as possible. In order to do so, an extensive vetting process is required to evaluate evidence and prioritize inclusion of genes likely to provide diagnostic results. Several groups have developed standardized CV scoring systems to systematically analyze published evidence supporting gene–disease relationships (Gonzalez‐Mantilla, Moreno‐De‐Luca, Ledbetter, & Martin, 2016; Rehm et al., 2015; Smith et al., 2017; Strande et al., 2017). These tools can help clinical laboratories identify the most clinically relevant genes to include on MGPTs.

Multiple lines of evidence should be considered for each gene, including the reported number of unrelated probands, the number of pathogenic variants identified (based on the laboratory's variant classification scheme), gene function, studies of model organisms, and other supporting evidence as applicable (Figure S1; Smith et al., 2017). Following review of all available evidence for a given gene–disease relationship, the relevant scoring criteria are weighed such that the score reflects the level of evidence of the strength of the gene–disease relationship.

Depending on the CV score, the gene–disease relationship is classified as either no evidence, limited, moderate, strong, or definitive evidence (Figure S1). Genes with a score of moderate or higher are considered “characterized”, while genes with lower scores are considered “candidate” genes. These scoring criteria can be used to interpret results from clinical diagnostic exome sequencing tests as well as for selecting genes for MGPTs. Inclusion of genes with lower CV scores on MGPTs may lead to uninformative clinical results for patients and may complicate results disclosures for clinicians (Eccles, Copson, Maishman, Abraham, & Eccles, 2015; Richter et al., 2013; Turbitt, Halliday, Amor, & Metcalfe, 2015). The purpose of this study is to determine the impact of including genes with limited CV for an association with hereditary cardiovascular‐related disease on the detection rate and VUS burden of MGPTs.

## 2 METHODS

### 2.1 Editorial policies and ethical considerations

Solutions Institutional Review Board determined this study to be exempt from the Office for Human Research Protections Regulations for the Protection of Human Subjects (45 CFR 46) under category 4.

### 2.2 Data analysis

To assess the impact of using an evidence‐based and standardized CV scoring system for panel design, and its impact on detection rate, we retrospectively reviewed data from 3,524 samples submitted to Ambry Genetics between March 2010 and December 2016 for genetic testing on one of the 13 different hereditary cardiovascular disease MGPT. The 13 MGPTs comprise a total of 106 genes associated with various types of arrhythmia, cardiomyopathy, hypercholesterolemia, and aortic aneurysm and dissection (see Supplementary methods). The scoring system developed by Smith et al. (2017) was used to rigorously assess the CV of the genes on these panels as of December 2016. The genes were categorized as having either limited, moderate, strong, or definitive CV scores. The detection rates of pathogenic or likely pathogenic variants (collectively referred to as VLP/P in the analysis below) were then compared among these four categories based on the clinical laboratory's classification scheme (Figure S1). In addition, there were a few genes with multiple CV scores depending on the associated phenotype; in this study we assumed the higher CV score for each gene as individual patient phenotype was not available in our dataset.

## 3 RESULTS

**Figure 1:**
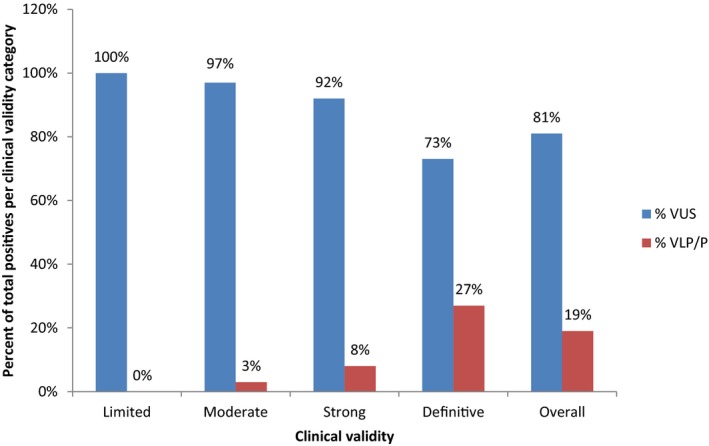
Bar graph showing perecent of total positive results per CV category. The rate of VUS is illustrated in blue, and VLP/P rate is shown in red. The rate of VLP/P increased with the score of CV

Among the 106 genes included on at least one of 13 hereditary cardiovascular disease MGPTs, 88% are considered to be characterized, with 42% classified as definitive, 17% as strong, and 29% as moderate. The remaining 12% were found to have limited CV (Table 1). Of the 3,524 total patients tested, 17% (*n* = 597) were positive with at least one VLP/P, 38% (*n* = 1,351) had only VUS, and 45% (*n* = 1,576) were negative. Overall, VLP/P variants represented 19% of the alterations reported among the 106 genes associated with cardiovascular disease, with VUS comprising the remaining 81% of reported calls (Figure S3). The proportion of VLP/P varied, however, when comparing genes across CV categories, with the rate of VLP/P increasing with the strength of the CV score (Figure 1). At least one VLP/P was found among 68% of those genes classified as definitive, 67% of genes classified as strong, and 29% of genes classified as moderate, while no VLP/P findings were identified among genes with limited evidence of CV. Furthermore, the rate of VLP/P among total reportable variants per CV category was 27% (range per gene: 0%–82%) for genes classified as definitive, 8% (range per gene: 0%–83%) for strong, 3% (range per gene: 0%–33%) for moderate and 0% for limited (Figure 1; Table 1; Tables S1–S3). Alternatively, the rate of VUS per CV category was 77% for definitive genes, 83% for strong genes, 95% for moderate genes, and 100% for limited genes (Figure 1; Table 1). The VUS identified in limited genes accounted for 7.8% (*n* = 191) of the total VUS reported.

**Table 1:** Distribution of VUS and VLP/P rates per clinical validity category

	Definitive	Strong	Moderate	Limited	Overall
Total genes, n (% of total)	44 (42)	17 (17)	31 (29)	13 (12)	106 (100)
Total VUS, n (% of all reported variants)	1,405 (73)	348 (92)	510 (97)	191 (100)	2,454 (81)
Average VUS per gene, n	32	19	17	14	23
Total VLP/P n, (% of all reported variants)	528 (27)	31 (8)	15 (3)	0 (0)	575 (19)
Average VLP/P, n	12	1.8	0.5	0	5
Genes with at least 1 VLP/P, n (%)	30 (68)	12 (67)	9 (29)	0 (0)	51 (48)
Genes with no VLP/P, n (%)	14 (32)	6 (33)	22 (71)	13 (100)	55 (52)

At least one VLP/P was identified in 51 out of 106 genes, while 55 had no pathogenic findings in our cohort (Table 1; Table S1–S3). Among genes with at least one VLP/P, 60% (*n* = 30) were classified as definitive, 24% (*n* = 12) were strong, and 16% (*n* = 9) were moderate. The highest number of VLP/Ps (*n* = 94) were reported in both *MYBPC3* (MIM: 600,958; definitive for hypertrophic and dilated cardiomyopathies[HCM/DCM]) and *FBN1* (MIM: 134,797; definitive for Marfan syndrome), accounting for 60% and 51% of the total reported findings for these genes, respectively (Table S1–S3). No genes with a limited CV score were found to have a VLP/P finding.

Three genes—*CALM1 *(*MIM: 114,180*), *KCNJ5* (*MIM: 600,734*), and *LDB3* (*MIM: 605,906*)—had neither pathogenic variants nor VUS reported. *CALM1* and *KCNJ5 *have strong and limited CV scores for long QT syndrome, respectively, and *LDB3* scored moderate for DCM and left ventricular non‐compaction (LVNC). The remaining 103 genes had at least one VUS identified. The highest number of VUS (*n* = 285) was reported in titin (*TTN (MIM: 188,840*); definitive for DCM), which accounted for 89% of the total reported calls for this gene (Table S1–S3). While titin (*TTN*) is classified as definitive for DCM, it is widely known to have a high VUS rate due to its large size, resulting in greater accumulation of missense alterations (Chauveau, Rowell, & Ferreiro, 2014). The mechanism of disease for *TTN* is loss of function. Therefore, truncating and frameshift variants are typically deleterious while missense variants are more difficult to classify without multiple additional lines of evidence. Interestingly, when comparing the results of the most comprehensive cardiovascular disease panel in this dataset ordered with or without the addition of *TTN *(CardioNext ± TTN, either 85/84 genes), the percent yield did not differ: 9% of all findings were VLP/P and 91% were VUS in both groups, suggesting that while *TTN* may increase the VUS rate, there is a concomitant increase in diagnostic yield as well. Correspondingly, the total number of VLP/P and VUS reported among the 101 cases tested without *TTN* were lower (14 and 77, respectively), compared to those reported among the 106 cases tested with *TTN* (27 and 171, respectively).

## 4 DISCUSSION

Detection rate and VUS burden appear to be influenced by several factors, including the specific phenotype, the number of genetic contributors to the phenotype, and panel size. In general, larger panels may return more positive pathogenic findings, however, they can also increase VUS burden (Pugh et al., 2014). In an assessment of DCM patients undergoing genetic testing by Pugh et al. (2014), detection rate was observed to increase with panel size; only 10% of patients undergoing a five‐gene MGPT had positive results, while 37% had positive findings when undergoing a 46‐gene MGPT. With this increase in detection rate, there was a respective increase of VUS burden from 4.6% to 51% (Pugh et al., 2014). However, the findings from our data suggest that the bulk of the increase in positive pathogenic findings would likely come from genes with a definitive, strong, or moderate CV while those genes with limited CV would likely only add uncertain findings.

Furthermore, a recent study by Ouellette et al. (2017) showed an increase in pathogenic findings when utilizing a phenotype‐targeted panel, as opposed to a large pan‐cardio panel, in a cohort of pediatric cardiomyopathy patients (Ouellette et al., 2017). When testing in this cohort was restricted to a phenotype‐targeted panel of 5–24 relevant, well‐established genes, positive pathogenic findings were detected in 32% of individuals, while VUS results were returned in 30% of patients (Ouellette et al., 2017). This result is compared in this same study to a large pan‐cardio panel consisting of 46–72 genes, where only 15% of tested individuals had a positive result, but 87% were found to have a VUS (Ouellette et al., 2017). In fact, 29 of the genes on the pan‐cardio panel in the Ouellette et al. (2017) study had no pathogenic findings returned in any patient, either because they lacked relevance to the patient's phenotype, or because of a very limited CV association with *any* cardiac phenotype (per our own assessment of CV for these genes). Therefore, excluding genes of limited CV may help limit the VUS burden without limiting the detection rate of the panel, as 100% of findings in limited genes in our data set were VUS results. Further research comparing the detection rates and VUS burden between multiple phenotype‐targeted panels in large cohorts could help clarify whether restricting panels to clinically validated and phenotypically relevant genes would increase detection rates without substantially increasing VUS burden. Of note, while there were no pathogenic findings detected in any of the limited genes assessed, there were also 32% of definitive genes, 33% of strong genes, and 71% of moderate genes with no pathogenic or likely pathogenic results. These results may be explained by a number of factors. Even in well‐established gene–disease relationships, mechanism of disease can be dependent on variant type or location within the gene. It is difficult or impossible to interpret pathogenicity without functional analysis of alterations, which are often not available for novel variants. Variant interpretation may be further impaired by insufficient clinical data, precluding accurate correlation between the patient's clinical presentation and genetic results. In addition, the establishment of a gene–disease relationship as characterized, rather than limited, does not quantify the proportion of a given gene's contribution to disease. Some of these characterized genes may have no positive findings because they are exceedingly rare, although evidentially well‐supported contributors to disease. The wealth of scientific evidence for these genes justifies their existence on an MGPT, on the rare chance that a result may be identified in a patient.

These results are most important to consider in the context of the impact to both the patient, who ultimately stands to benefit the most from evidence‐driven panel design, and the clinician, who is responsible for disclosing the results in an understandable manner. A survey of genetic counselors’ confidence regarding VUS results showed that while most counselors are confident in their own ability to explain and understand a VUS result, they are less confident in discussing possible clinical management options for these results, and in the patient's ability to understand the results (Scherr, Lindor, Malo, Couch, & Vadaparampil, 2015). Indeed, many patients, or parents of patients, show poor recall of terminology surrounding VUS results, and incorrectly interpret the results as “positive” regardless of the skill of the clinician providing the results (Kiedrowski, Owens, Yashar, & Schuette, 2015). While this research was framed specifically around *BRCA1/2* testing and chromosomal microarray analysis, it is important to consider that potentially increasing VUS burden without increasing pathogenic findings could result in similar concerns within the context of a cardiovascular genetics clinic.

There are a few limitations to this study. The inclusion of larger‐sized genes in the available MGPTs increases the likelihood of higher VUS rates, even in genes with definitive CV (such as *TTN*). Some genes included on MGPTs have an unclear mechanism of disease and a limited number of functional studies, which make it challenging to interpret and determine pathogenicity during variant assessment. This could potentially lead to a higher number of VUS and lower detection rates overall, even for genes with more convincing evidence and higher CV scores. Additionally, ordering patterns were not equal across all panel types; some sub‐panels were ordered more frequently than others, resulting in variable numbers of patients being tested for each gene (Tables S1). Furthermore, since results were assessed based on the phenotype for which each gene received the highest CV score, and because we did not have access to detailed phenotype data for every patient in this cohort, interpretation of results could not be broken down into multiple phenotypes and their associated gene's CV score. Finally, CV scores are dependent on variant classification, and vice versa. This circular logic biases the significance of a gene with a lower CV leading to a lower likelihood of finding a VLP/P.

The ultimate objective of genetic testing is to find answers for patients to help guide their medical management, and to provide preventative care for family members who are not yet symptomatic. VUS may be difficult for patients to understand, and cannot be used to guide medical management decisions. Designing panels to limit these types of findings will maximize the ability to return a positive result, resulting in a more informed and less anxious patient, without placing additional burden on the clinician to interpret VUS findings in genes that have limited evidence of CV. When designing MGPTs, an evidence‐based evaluation of CV emphasizes inclusion of genes with strong gene–disease relationships while de‐emphasizing inclusion of genes with limited gene‐disease relationships.

## CONFLICT OF INTEREST

All authors are full‐time salaried employees of Ambry Genetics or were full‐time employees at the time this manuscript was drafted. Multi‐gene panel testing comprises the majority of Ambry's test menu.

## References

[mgg3630-bib-0001] Boycott, K. M. , Rath, A. , Chong, J. X. , Hartley, T. , Alkuraya, F. S. , Baynam, G. , … Lochmüller, H. (2017). International cooperation to enable the diagnosis of all rare genetic diseases. American Journal of Human Genetics, 100(5), 695–705. 10.1016/j.ajhg.2017.04.003 28475856 PMC5420351

[mgg3630-bib-0002] Chauveau, C. , Rowell, J. , & Ferreiro, A. (2014). A rising titan: *TTN* review and mutation Update. Human Mutation, 35(9), 1046–1059. 24980681 10.1002/humu.22611

[mgg3630-bib-0003] Chong, J. X. , Buckingham, K. J. , Jhangiani, S. N. , Boehm, C. , Sobreira, N. , Smith, J. D. , … Bamshad, M. J. (2015). The genetic basis of Mendelian phenotypes: Discoveries, challenges, and opportunities. American Journal of Human Genetics, 97(2), 199–215. 10.1016/j.ajhg.2015.06.009 26166479 PMC4573249

[mgg3630-bib-0004] Eccles, B. K. , Copson, E. , Maishman, T. , Abraham, J. E. , & Eccles, D. M. (2015). Understanding of BRCA VUS genetic results by breast cancer specialists. BMC Cancer, 15, 936. 26608569 10.1186/s12885-015-1934-1 PMC4660681

[mgg3630-bib-0005] Ezkurdia, I. , Juan, D. , Rodriguez, J. M. , Frankish, A. , Diekhans, M. , Harrow, J. , … Tress, M. L. (2014). Multiple evidence strands suggest that there may be as few as 19 000 human protein‐coding genes. Human Molecular Genetics, 23(22), 5866–5878. 10.1093/hmg/ddu309 24939910 PMC4204768

[mgg3630-bib-0006] Ghouse, J. , Skov, M. W. , Bigseth, R. S. , Ahlberg, G. , Kanters, J. K. , & Olesen, M. S. (2017). Distinguishing pathogenic mutations from background genetic noise in cardiology: The use of large genome databases for genetic interpretation. Clinical Genetics, 93(3), 459–466. 10.1111/cge.13066 28589536

[mgg3630-bib-0007] Gonzalez‐Mantilla, A. J. , Moreno‐De‐Luca, A. , Ledbetter, D. H. , & Martin, C. L. (2016). A cross‐disorder method to identify novel candidate genes for developmental brain disorders. JAMA Psychiatry, 73(3), 275–283. 10.1001/jamapsychiatry.2015.2692 26817790 PMC5333489

[mgg3630-bib-0008] Kiedrowski, L. A. , Owens, K. M. , Yashar, B. M. , & Schuette, J. L. (2015). Parents’ perspectives on variants of uncertain significance from chromosome microarray analysis. Journal of Genetic Counseling 25(1), 101–111. 10.1007/s10897-015-9847-3 25983052

[mgg3630-bib-0009] Ouellette, A. C. , Mathew, J. , Manickaraj, A. K. , Manase, G. , Zahavich, L. , Wilson, J. , … … S. (2017). Clinical genetic testing pediatric cardiomyopathy: Is bigger better? Clinical Genetics. 10.1111/cge.13024. [Epub ahead of print]. 28369760

[mgg3630-bib-0010] Pugh, T. J. , Kelly, M. A. , Gowrisankar, S. , Hynes, E. , Seidman, M. A. , Baxter, S. M. , … Funke, B. H. (2014). The landscape of genetic variation in dilated cardiomyopathy as surveyed by clinical DNA sequencing. Genetics in Medicine, 16(8), 601–608. 10.1038/gim.2013.204 24503780

[mgg3630-bib-0011] Rehm, H. L. , Berg, J. S. , Brooks, L. D. , Bustamante, C. D. , Evans, J. P. , Landrum, M. J. , … Watson, M. S. (2015). ClinGen – The clinical genome resource. New England Journal of Medicine, 372, 2235–2242. 10.1056/NEJMsr1406261 26014595 PMC4474187

[mgg3630-bib-0012] Richards, S. , Aziz, N. , Bale, S. , Bick, D. , Das, S. , Gastier‐Foster, J. , … Rehm, H. L. (2015). Standards and guidelines for the interpretation of sequence variants: A joint consensus recommendation of the American College of Medical Genetics and Genomics and the Association for Molecular Pathology. Genetics in Medicine, 17(5), 405 10.1038/gim.2015.30 25741868 PMC4544753

[mgg3630-bib-0013] Richter, S. , Haroun, I. , Graham, T. C. , Eisen, A. , Kiss, A. , & Warner, E. (2013). Variants of unknown significance in BRCA testing: Impact on risk perception, worry, prevention and counseling. Annals of Oncology, 24(suppl_8), viii69‐viii74. 24131974 10.1093/annonc/mdt312

[mgg3630-bib-0014] Scherr, C. L. , Lindor, N. M. , Malo, T. L. , Couch, F. J. , & Vadaparampil, S. T. (2015). Genetic counselors’ practices and confidence regarding variant of uncertain significance results and reclassification from BRCA testing. Clinical Genetics 88(6), 523–529. 25640009 10.1111/cge.12563 PMC4522387

[mgg3630-bib-0015] Smith, E. D. , Radtke, K. , Rossi, M. , Shinde, D. N. , Darabi, S. , El‐Khechen, D. , … Farwell Hagman, K. D. (2017). Classification of genes: Standardized clinical validity assessment of gene‐disease associations aids diagnostic exome analysis and reclassifications. Human Mutation, 38(5), 600–608. 10.1002/humu.23183 28106320 PMC5655771

[mgg3630-bib-0016] Strande, N. T. , Riggs, E. R. , Buchanan, A. H. , Ceyhan‐Birsoy, O. , DiStefano, M. , Dwight, S. S. , & Wright, M. W. (2017). Evaluating the clinical validity of gene‐disease associations: An evidence‐based framework developed by the clinical genome resource. American Journal of Human Genetics, 100(6), 895–906. 28552198 10.1016/j.ajhg.2017.04.015 PMC5473734

[mgg3630-bib-0017] Turbitt, E. , Halliday, J. L. , Amor, D. J. , & Metcalfe, S. A. (2015). Preferences for results from genomic microarrays: Comparing parents and health care providers. Clinical Genetics, 87(1), 21–29. 10.1111/cge.12398 24773164

