# Supplementary material for: Clinical validity assessment of genes for inclusion in multi‐gene panel testing: A systematic approach
Source: Mol Genet Genomic Med. 2019 Mar 21;7(5):e630. doi: 10.1002/mgg3.630 (PMC6503028; doi:10.1002/mgg3.630)
Supplement: Supplementary file 1 [file MGG3-7-e630-s001.pdf]

Figure Legends

Figure S1. Clinical validity scoresheet showing multiple lines of evidence considered including number of unrelated patients reported, number of publications reporting independent probands, number of pathogenic variants based on the clinical laboratory’s classification scheme, gene function and disruption as well as available model organisms. Sum of the total points determines the clinical validity, from no evidence as the lowest score to definitive clinical validity with the strongest evidence.

Figure S2. Distribution of clinical validity score across the 106 genes associated with cardiovascular disease is illustrated in different colors.

Figure S3. Distribution of results reported comparing overall VUS rate to VLP/P rate among 106 genes associated with cardiovascular disease. VLP/P variants in red, comparing to VUS in blue.

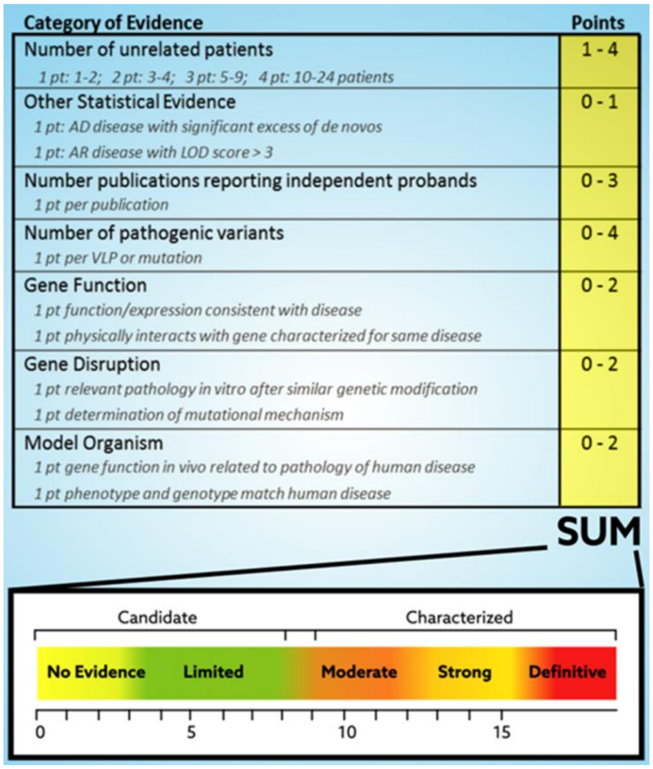

Adapted from Smith et al. (2017)

Figure S1. Clinical validity scoresheet

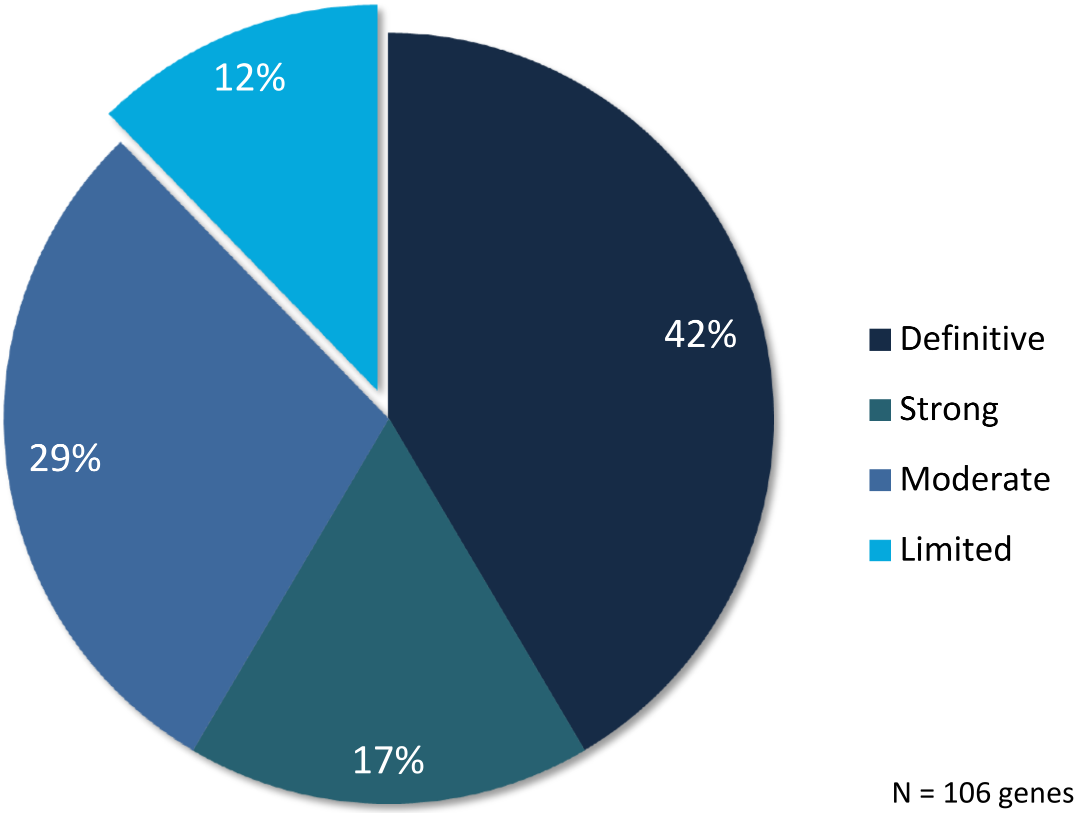

Figure S2. Distribution of Clinical Validity Scores

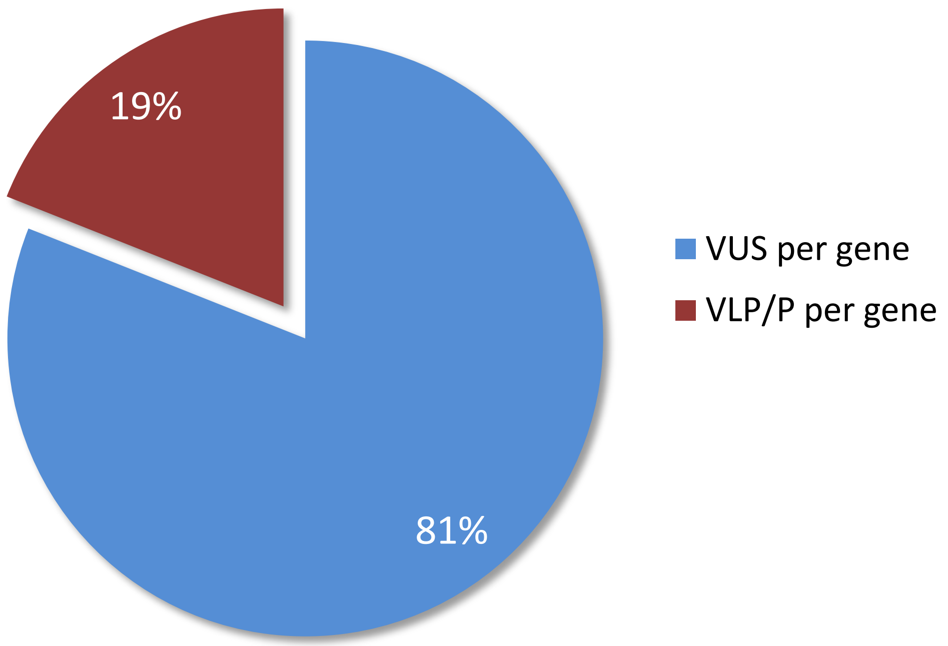

Figure S3. Distribution of Results Reported
