# Supplementary material for: Clinical validity assessment of genes for inclusion in multi‐gene panel testing: A systematic approach
Source: Mol Genet Genomic Med. 2019 Mar 21;7(5):e630. doi: 10.1002/mgg3.630 (PMC6503028; doi:10.1002/mgg3.630)
Supplement: Supplementary file 2 [file MGG3-7-e630-s002.docx]

Table S1:  **Summary of results for genes included on hereditary cardiovascular multi-gene panel tests (MGPT). Listed alphabetically by gene.**

| *Gene* | GenBank Reference Sequence | | | | | Gene MIM ID | | | Total Cases | | % VLP/P | N VLP/P | % VUS | N VUS | Highest CV score^a^ | Phenotype Scored as highest CV^b^ | References (PMID) | ClinGen Gene-Disease Validity Classification^c^ |
| --- | --- | --- | --- | --- | --- | --- | --- | --- | --- | --- | --- | --- | --- | --- | --- | --- | --- | --- |
| *ABCC9* | NM_005691.2 | | | | | 601439 | | | 587 | | 0% | 0 | 100% | 7 | Moderate | DCM | 15034580 | Not scored for DCM |
| *ACTA2* | NM_001613.2 | | | | | 102620 | | | 1587 | | 40% | 6 | 60% | 9 | Definitive | TAAD | 17994018 19409525 | Definitive |
| *ACTC1* | NM_005159.4 | | | | | 102540 | | | 1134 | | 0% | 0 | 100% | 7 | Strong | HCM | 22464770 9563954 10494087 | Definitive |
| *ACTN2* | NM_001103.2 | | | | | 102573 | | | 1097 | | 5% | 1 | 95% | 19 | Moderate | HCM | 20022194 25173926 22253474 | Moderate |
| *AKAP9* | NM_005751.4 | | | | | 604001 | | | 880 | | 0% | 0 | 100% | 50 | Limited | LQTS | 18093912 11799244 | Limited |
| *ANK2* | NM_001148.4 | | | | | 106410 | | | 912 | | 2% | 1 | 98% | 50 | Strong | LQTS | 15178757 12571597 22586405 17242276 | Not scored for LQTS (Disputed for Brugada) |
| *ANKRD1* | NM_014391.2 | | | | | 609599 | | | 1097 | | 0% | 0 | 100% | 12 | Moderate | HCM | 19608031 23572067 | Limited |
| *BAG3* | NM_004281.3 | | | | | 603883 | | | 587 | | 27% | 4 | 73% | 11 | Strong | DCM | 21898660 21353195 21459883 | Not scored for DCM |
| *CACNA1C* | NM_000719.6 | | | | | 114205 | | | 880 | | 0% | 0 | 100% | 20 | Moderate | LQTS | 17224476 15655131 20817017 | Not scored for LQTS (Disputed for Brugada) |
| *CACNA2D1* | NM_000722.2 | | | | | 114204 | | | 859 | | 0% | 0 | 100% | 13 | Moderate | BrS | 20817017 19429829 25527503 | Disputed |
| *CACNB2* | NM_201590.2 | | | | | 600003 | | | 859 | | 0% | 0 | 100% | 23 | Moderate | BrS | 17224476 19358333 22840528 | Disputed |
| *CALM1* | NM_006888.4 | | | | | 114180 | | | 891 | | n/a | 0 | n/a | 0 | Strong | LQTS | 23388215  24076290 27374306 | Not scored |
| *CASQ2* | NM_001232.3 | | | | | 114251 | | | 891 | | 33% | 4 | 67% | 8 | Moderate | CPVT | 16908766 11704930 27157848 11401939 | Not scored for CPVT |
| *CAV3* | NM_033337.2 | | | | | 601253 | | | 880 | | 0% | 0 | 100% | 2 | Moderate | LQTS | 17060380 17275750 17210839 | Not scored |
| *CBS* | NM_000071.2 | | | | | 613381 | | | 1587 | | 0% | 0 | 100% | 12 | Definitive | Homocystinuria (TAAD) | 18280597 12552044 20142522 15087459 | Not scored |
| *COL3A1* | NM_000090.3 | | | | | 120180 | | | 1587 | | 31% | 14 | 69% | 31 | Definitive | EDS (TAAD) | 21637106 11577371 9399899 9712532 25758994 | Definitive |
| *COL5A1* | NM_000093.4 | | | | | 120215 | | | 1587 | | 4% | 3 | 96% | 73 | Definitive | EDS (TAAD) | 23587214 16278879 9042913 | Not scored |
| *COL5A2* | NM_000393.3 | | | | | 120190 | | | 1587 | | 2% | 1 | 98% | 51 | Strong | EDS (TAAD) | 23587214 16278879 22696272 | Not scored |
| *CRYAB* | NM_001885.1 | | | | | 123590 | | | 473 | | 0% | 0 | 100% | 4 | Moderate | Myofibrillar myopathy (CM) | 16483541 16793013 | Not scored |
| *CSRP3* | NM_003476.3 | | | | | 600824 | | | 1097 | | 0% | 0 | 100% | 4 | Moderate | HCM | 12507422 14567970 9039266 | Moderate |
| *DES* | NM_001927.3 | | | | | 125660 | | | 587 | | 0% | 0 | 100% | 9 | Definitive | DCM | 23143191 10430757 17221859 20718792 22153487 | Not scored for DCM |
| *DMD* | NM_004006.2 | | | | | 300377 | | | 587 | | 9% | 4 | 91% | 39 | Definitive | DMD/BMD (DCM) | 9170393 9170407 8504498 29801751 27230049 12359139 | Not scored |
| *DSC2* | NM_024422.3 | | | | | 125645 | | | 1144 | | 3% | 1 | 97% | 34 | Definitive | ARVD | 17186466 17033975 23824749 | Not scored |
| *DSG2* | NM_001943.3 | | | | | 125671 | | | 1144 | | 10% | 3 | 90% | 27 | Definitive | ARVD | 23824749 16505173 20400443 17105751 16773573 | Not scored |
| *DSP* | NM_004415.2 | | | | | 125647 | | | 1144 | | 17% | 9 | 83% | 44 | Definitive | ARVD | 23824749 22426942 23468208 20864495 12373648 16917092 | Not scored for ARVD |
| *EMD* | NM_000117.2 | | | | | 300384 | | | 473 | | 0% | 0 | 100% | 1 | Definitive | EMD (CM) | 11973618 10838246 7894480 | Not scored |
| *EYA4* | NM_004100.4 | | | | | 603550 | | | 587 | | 0% | 0 | 100% | 6 | Limited | DCM | 15735644 | Not scored for DCM |
| *FBN1* | NM_000138.4 | | | | | 134797 | | | 1587 | | 51% | 94 | 49% | 89 | Definitive | Marfan sx (TAAD) | 16571647 17492313 20591885 15241795 | Definitive |
| *FBN2* | NM_001999.3 | | | | | 612570 | | | 1587 | | 4% | 3 | 96% | 78 | Definitive | Congenital contractural arachnodactyly (TAAD) | 7493032 11754102 19006240 | Not scored for Congenital contractural arachnodactyly |
| *FKTN* | NM_001079802.1 | | | | | 607440 | | | 587 | | 0% | 0 | 100% | 15 | Limited | DCM | 17036286 19015585 | Not scored |
| *FLNA* | NM_001456.3 | | | | | 300017 | | | 1587 | | 7% | 3 | 93% | 38 | Definitive | Periventricular heterotopia (TAAD) | 29334594 26188975 17172441 22121117 | Not scored for Periventricular heterotopia |
| *FXN* | NM_000144.4 | | | | | 606829 | | | 983 | | 0% | 0 | 100% | 4 | Definitive | Friedreich ataxia (HCM) | 22764179  11159298 | Definitive |
| *GATA4* | NM_002052.3 | | | | | 600576 | | | 267 | | 0% | 0 | 100% | 7 | Definitive | Congenital heart defects | 23626780  18393291 | Not scored |
| *GATAD1* | NM_021167.3 | | | | | 614518 | | | 473 | | 0% | 0 | 100% | 5 | Limited | DCM | 21965549 | Not scored |
| *GLA* | NM_000169.2 | | | | | 300644 | | | 983 | | 43% | 3 | 57% | 4 | Definitive | Fabry disease (HCM) | 12585833 16227523 11914245 | Definitive |
| *GPD1L* | NM_015141.3 | | | | | 611778 | | | 859 | | 20% | 1 | 80% | 4 | Moderate | BrS | 17967977 17967976 11839626 19666841 18762705 | Disputed |
| *HCN4* | NM_005477.2 | | | | | 605206 | | | 859 | | 0% | 0 | 100% | 34 | Limited | BrS | 22840528 19165230 | Disputed |
| *JAG1* | NM_000214.2 | | | | | 601920 | | | 267 | | 0% | 0 | 100% | 3 | Definitive | Alagille sx (congenital heart defects) | 21934706 14684686  9207787  9207788 | Not scored |
| *JPH2* | NM_020433.4 | | | | | 605267 | | | 983 | | 0% | 0 | 100% | 21 | Moderate | HCM | 17509612 28393127 21216834 17476457 15541368 | Moderate |
| *JUP* | NM_002230.2 | | | | | 173325 | | | 1144 | | 0% | 0 | 100% | 17 | Moderate | ARVD | 17924338 25765472 24125834  18937352 | Not scored |
| *KCND3* | NM_004980.4 | | | | | 605411 | | | 859 | | 0% | 0 | 100% | 5 | Moderate | BrS | 22457051 21349352 22840528 22284586 26016905 10200233 | Disputed |
| *KCNE1* | NM_000219.3 | | | | | 176261 | | | 880 | | 44% | 7 | 56% | 9 | Definitive | LQTS | 11799244 9354802 16922724 10973849 15051636 | Moderate for Jervell and Lange-Nielsen syndrome 2 |
| *KCNE2* | NM_172201.1 | | | | | 603796 | | | 880 | | 0% | 0 | 100% | 5 | Strong | LQTS | 16922724 10973849 15840476 | Not scored |
| *KCNE3* | NM_005472.4 | | | | | 604433 | | | 859 | | 0% | 0 | 100% | 4 | Limited | BrS | 19122847 | Disputed |
| *KCNH2* | NM_000238.3 | | | | | 152427 | | | 932 | | 54% | 37 | 46% | 31 | Definitive | LQTS | 7889573 16922724 9509262 10898405 11854117 9694858 | Not scored for LQTS (Disputed for Brugada) |
| *KCNJ2* | NM_000891.2 | | | | | 600681 | | | 912 | | 29% | 2 | 71% | 5 | Definitive | Andersen-Tawil sx (LQTS) | 12148092 17324964 16217063 12796536 12086641 12163457 16419128 | Not scored |
| *KCNJ5* | NM_000890.3 | | | | | 600734 | | | 267 | | n/a | 0 | n/a | 0 | Limited | LQTS | 20560207 23872692 24574546 | Not scored |
| *KCNJ8* | NM_004982.2 | | | | | 600935 | | | 859 | | 0% | 0 | 100% | 3 | Limited | BrS | 20558321 22056721 22840528 21836131 | Disputed |
| *KCNQ1* | NM_000218.2 | | | | | 607542 | | | 932 | | 59% | 44 | 41% | 30 | Definitive | LQTS | 16922724 25344363 9753711  15051636 8528244 15840476 10973849 | Definitive for Jervell and Lange-Nielsen syndrome |
| *LAMA4* | NM_002290.3 | | | | | 600133 | | | 587 | | 0% | 0 | 100% | 24 | Moderate | DCM | 17646580 16204254 | Not scored |
| *LAMP2* | NM_002294.2 | | | | | 309060 | | | 1097 | | 0% | 0 | 100% | 5 | Definitive | Danon disease (HCM & DCM) | 15673802 15889279 21415759 15253947 19318653 17899313  22695892 | Definitive |
| *LDB3* | NM_007078.2 | | | | | 605906 | | | 624 | | n/a | 0 | n/a | 0 | Moderate | Myofibrillar myopathy (DCM & LVNC) | 14662268 19028670 14660611 | Not scored |
| *LMNA* | NM_005572.3 | | | | | 150330 | | | 1281 | | 43% | 13 | 57% | 17 | Definitive | DCM | 12673789 10580070 12920062 29175975 27576561 21810905 16601451 | Not scored |
| *MED12* | NM_005120.2 | | | | | 300188 | | | 1587 | | 0% | 0 | 100% | 16 | Moderate | Lujan-Fryns sx (TAAD) | 17036352 10508979 17369503 | Definitive for MED12-related intellectual disability syndrome |
| *MYBPC3* | NM_000256.3 | | | | | 600958 | | | 1160 | | 60% | 94 | 40% | 63 | Definitive | HCM & DCM | 7493025 12707239 22057632  9562578 11499719 | Definitive |
| *MYH11* | NM_002474.2 | | | | | 160745 | | | 1587 | | 1% | 1 | 99% | 81 | Strong | TAAD | 16444274 17666408 18544034 | Definitive |
| *MYH6* | NM_002471.3 | | | | | 160710 | | | 1097 | | 0% | 0 | 100% | 64 | Moderate | HCM & DCM | 15998695 11815426 | Limited for HCM |
| *MYH7* | NM_000257.2 | | | | | 160760 | | | 1160 | | 42% | 43 | 58% | 59 | Definitive | HCM | 19864899 19808347 8981935 12974739 1975517 24113344 | Definitive |
| *MYL2* | NM_000432.3 | | | | | 160781 | | | 983 | | 36% | 4 | 64% | 7 | Definitive | HCM | 9535554 12404107 26074085 12707239 | Definitive |
| *MYL3* | NM_000258.2 | | | | | 160790 | | | 983 | | 56% | 5 | 44% | 4 | Strong | HCM | 22957257 12021217 8673105 23594557 | Definitive |
| *MYLK* | NM_053025.3 | | | | | 600922 | | | 1587 | | 3% | 2 | 97% | 63 | Moderate | TAAD | 21055718 28401540 29544503 | Strong |
| *MYOZ2* | NM_016599.4 | | | | | 605602 | | | 983 | | 0% | 0 | 100% | 1 | Limited | HCM | 17347475 22987565 | Limited |
| *MYPN* | NM_032578.2 | | | | | 608517 | | | 1097 | | 0% | 0 | 100% | 42 | Definitive | DCM | 22286171 18006477 22892539 | Not scored for DCM |
| *NEXN* | NM_144573.3 | | | | | 613121 | | | 983 | | 17% | 3 | 83% | 15 | Moderate | DCM | 19881492 | Not scored for DCM |
| *NKX2-5* | NM_004387.3 | | | | | 600584 | | | 942 | | 0% | 0 | 100% | 15 | Definitive | Congenital heart defects | 25503402 28690296 27855642 | Not scored |
| *NOTCH1* | NM_017617.3 | | | | | 190198 | | | 1587 | | 0% | 0 | 100% | 97 | Moderate | BAV/TAAD | 25907466 16729972 17662764 23102684 16025100 23578328 18593716 20951801 26164125 25260786 | Limited |
| *PKP2* | NM_004572.3 | | | | | 602861 | | | 1144 | | 37% | 21 | 63% | 36 | Definitive | ARVD | 22426942 16549640 15489853 15479741 | Definitive |
| *PLN* | NM_002667.3 | | | | | 172405 | | | 1097 | | 83% | 5 | 17% | 1 | Strong | DCM | 22155237 16432188 12639993 12610310 | Definitive (cardiomyopathy) |
| *PLOD1* | NM_000302.3 | | | | | 153454 | | | 1587 | | 0% | 0 | 100% | 32 | Definitive | EDS (TAAD) | 15666309 21699693 28306225 28306229 28981071 | Not scored for EDS |
| *PRKAG2* | NM_016203.3 | | | | | 602743 | | | 288 | | 27% | 3 | 73% | 8 | Strong | HCM | 15673802  11371514 18195183 26729852 | Definitive |
| *PRKG1* | NM_006258.3 | | | | | 176894 | | | 1587 | | 6% | 1 | 94% | 17 | Moderate | TAAD | 23910461 27442293 | Strong |
| *PTPN11* | NM_002834.3 | | | | | 176876 | | | 983 | | 56% | 5 | 44% | 4 | Definitive | Noonan sx (HCM) | 15673802  11371514 18195183 26729852 | Definitive |
| *RAF1* | NM_002880.3 | | | | | 164760 | | | 1097 | | 17% | 2 | 83% | 10 | Strong | Noonan sx (HCM) | 20052757 17603483 17603482 21396583 | Definitive |
| *RBM20* | NM_001134363.1 | | | | | 613171 | | | 587 | | 0% | 0 | 100% | 19 | Strong | DCM | 20590677 19712804 | Not scored |
| *RYR2* | NM_001035.2 | | | | | 180902 | | | 1176 | | 7% | 6 | 93% | 84 | Definitive | CPVT | 28237968 16272262 11208676 11157710 | Definitive |
| *SCN1B* | NM_001037.4 | | | | | 600235 | | | 859 | | 33% | 1 | 67% | 2 | Moderate | BrS | 18464934 29758173 25253298 22155597 26179811 22155598 28217227 | Disputed |
| *SCN2B* | NM_004588.4 | | | | | 601327 | | | 267 | | 0% | 0 | 100% | 1 | Moderate | BrS/AF | 23559163 26173111  26179811 19808477 27932425 24144883 | Disputed |
| *SCN3B* | NM_018400.3 | | | | | 608214 | | | 859 | | 0% | 0 | 100% | 4 | Moderate | BrS/AF | 20031595 21051419 23257389 24529773 20226894 20042427 11744748 | Disputed |
| *SCN4B* | NM_174934.3 | | | | | 608256 | | | 880 | | 0% | 0 | 100% | 10 | Limited | LQTS | 17592081 | Limited |
| *SCN5A* | NM_198056.2 | | | | | 600163 | | | 1252 | | 27% | 27 | 73% | 74 | Definitive | LQTS & BrS | 10590249 12417563 10220144 18451998 16922724 7889574 12736279 | Definitive |
| *SKI* | NM_003036.3 | | | | | 164780 | | | 1587 | | 0% | 0 | 100% | 21 | Definitive | Shprintzen-Goldberg sx (TAAD) | 23023332 15884042 24736733 | Definitive |
| *SLC2A10* | NM_030777.3 | | | | | 606145 | | | 1587 | | 0% | 0 | 100% | 16 | Definitive | Arterial tortuosity sx (TAAD) | 16550171 17935213  26376865 25373504 | Not scored for arterial tortuosity (limited for TAAD) |
| *SMAD3* | NM_005902.3 | | | | | 603109 | | | 1587 | | 30% | 6 | 70% | 14 | Definitive | LDS (TAAD) | 29392890 21217753 21778426 22167769 22633655 | Definitive |
| *SMAD4* | NM_005359.5 | | | | | 600993 | | | 978 | | 29% | 2 | 71% | 5 | Definitive | JPS-HHT (TAAD) | 16613914 15031030 17873119 20101697 22331366 | Definitive |
| *SNTA1* | NM_003098.2 | | | | | 601017 | | | 880 | | 0% | 0 | 100% | 21 | Limited | LQTS | 18591664 19684871 20009079 | Not scored |
| *TAZ* | NM_000116.3 | | | | | 300394 | | | 514 | | 50% | 1 | 50% | 1 | Definitive | Barth sx (CM) | 21947198 9345098 21068380 15098233 23398819 23843353 | Not scored |
| *TBX1* | NM_080647.1 | | | | | 602054 | | | 267 | | 0% | 0 | 100% | 3 | Definitive | Congenital heart defects | 11748311  14585638 17273972 | Not scored |
| *TBX20* | NM_001077653.2 | | | | | 606061 | | | 478 | | 0% | 0 | 100% | 5 | Strong | DCM | 26118961 17668378 27510170 26895318 | Not scored |
| *TBX5* | NM_000192.3 | | | | | 601620 | | | 731 | | 0% | 0 | 100% | 8 | Definitive | Holt-Oram sx | 16183809 8988165 25680289 8988165 11572777 | Not scored |
| *TCAP* | NM_003673.3 | | | | | 604488 | | | 1097 | | 0% | 0 | 100% | 8 | Moderate | HCM & DCM | 15582318 16352453 15582318 12507422 | Limited (HCM only) |
| *TGFB2* | NM_003238.3 | | | | | 190220 | | | 1587 | | 14% | 3 | 86% | 18 | Strong | LDS (TAAD) | 22772371 22772368 25163805  29392890 | Definitive |
| *TGFB3* | NM_003239.2 | | | | | 190230 | | | 2122 | | 4% | 1 | 96% | 22 | Moderate | LDS (TAAD) | 29392890 26184463 25835445 23824657 | Limited (TAAD only) |
| *TGFBR1* | NM_004612.2 | | | | | 190181 | | | 1587 | | 4% | 1 | 96% | 23 | Strong | LDS (TAAD) | 15731757 16928994 24443023 24355923 22414221 | Definitive |
| *TGFBR2* | NM_003242.5. | | | | | 190182 | | | 1587 | | 19% | 5 | 81% | 22 | Definitive | LDS (TAAD) | 16928994 15731757 24793577 24443023 | Definitive |
| *TMEM43* | NM_024334.2 | | | | | 612048 | | | 1144 | | 0% | 0 | 100% | 14 | Moderate | ARVD | 21636032 22725725 21214875 8313022 23812740 | Not scored |
| *TMPO* | NM_003276.2 | | | | | 188380 | | | 587 | | 0% | 0 | 100% | 5 | Limited | DCM | 16247757 | Refuted |
| *TNNC1* | NM_003280.2 | | | | | 191040 | | | 1097 | | 0% | 0 | 100% | 2 | Moderate | DCM | 15542288 15923195 21832052 20215591 | Moderate |
| *TNNI3* | NM_000363.4 | | | | | 191044 | | | 1097 | | 29% | 4 | 71% | 10 | Definitive | HCM | 15607392 9241277 21533915 12707239 | Definitive |
| *TNNT2* | NM_001001430.1 | | | | | 191045 | | | 1134 | | 72% | 21 | 28% | 8 | Definitive | HCM & DCM | 18651846  8205619 7898523  28840316 22144547 10085122 | Definitive |
| *TPM1* | NM_001018005.1 | | | | | 191010 | | | 1134 | | 33% | 4 | 67% | 8 | Strong | HCM & DCM | 9060904 7898523 10400910 25548289 | Definitive |
| *TRDN* | NM_006073.2 | | | | | 603283 | | | 891 | | 0% | 0 | 100% | 19 | Strong | CPVT | 22422768 19383796 26200674 23396608 19843516 | Not scored |
| *TRPM4* | NM_017636.3 | | | | | 606936 | | | 859 | | 3% | 1 | 97% | 28 | Strong | BrS/heart block | 19726882 20562447 21887725 24721656 29568272 | Disputed |
| *TTN* | NM_003319.4 | | | | | 188840 | | | 443 | | 11% | 36 | 89% | 285 | Definitive | DCM | 11788824 22335739 26315439 11846417 29238064 | Not scored for DCM |
| *TTR* | NM_000371.3 | | | | | 176300 | | | 940 | | 82% | 9 | 18% | 2 | Definitive | Amyloidosis | 23797140 28102864 27188913 27386769 20479782 | Definitive |
| *TXNRD2* | NM_006440.3 | | | | | 606448 | | | 587 | | 0% | 0 | 100% | 15 | Limited | DCM | 21247928 | Not scored |
| *VCL* | NM_014000.2 | | | | | 193065 | | | 1097 | | 0% | 0 | 100% | 19 | Moderate | DCM | 11815424 20474083 16236538 15331426 | Not scored for DCM |
|  |  | | | | |  | | |  | |  |  |  |  |  |  |  |  |
|  | |  | |  | | |  |  |  |  |  |  |  |  |  |  |  |  |
|  |  | |  | |  | | |  | |  |  |  |  |  |  |  |  |  |

^a^ Gene-disease relationships assessed as of December 2017, by method described in Smith *et al*., 2017

^b^ Table reflects the phenotype with the highest CV; many genes are associated with additional indications not listed here.

^c^ *https://search.clinicalgenome.org/kb/gene-validity* Accessed December 2018

Legend:
*VLP/P –* Variant likely pathogenic/pathogenic
*VUS* – Variant of uncertain significance

*CV* – Clinical Validity
*Sx –* Syndrome
*HCM –* Hypertrophic Cardiomyopathy
*DCM –* Dilated cardiomyopathy
*LQTS –* Long-QT Syndrome
*TAAD –* Thoracic aortic aneurysm and dissection
*ARVD –* Arrythmogenic Right-Ventricular Dysplasia
*EDS –* Ehlers Danlos Syndrome
*LDS –* Loeys-Dietz Syndrome
*BrS –* Brugada Syndrome
*DMD/BMD –* Duchenne/Becker Muscular Dystrophy
*CPVT –* Catecholaminergic Polymorphic Ventricular Tachycardia
*LVNC* – Left-ventricular Non-compaction
*JPS-HHT* – Juvenile Polyposis Syndrome – Hereditary Hemorrhagic Telangiectasia
*AF* – Atrial Fibrillation
