# Supplementary material for: Clinical validity assessment of genes for inclusion in multi‐gene panel testing: A systematic approach
Source: Mol Genet Genomic Med. 2019 Mar 21;7(5):e630. doi: 10.1002/mgg3.630 (PMC6503028; doi:10.1002/mgg3.630)
Supplement: Supplementary file 5 [file MGG3-7-e630-s005.docx]

**Supplemental Methods**

A retrospective analysis was performed on the data from 3,524 total cases tested for as many as 106 genes from a suite of cardiovascular MGPTs.

The tests include ARVDNext (n=79), CardioNext -TTN (n = 101), CardioNext +TTN (n = 166), CMNext - TTN (n = 43), CMNext + TTN (n = 163), CPVTNext (n=32), DCMNext (n=114), HCMFirst (n=27), HCMNext (n=510), LVNCNext (n=37), RhythmFirst (n=73), RhythmNext (n=592), TAADNext (n=1587). Full gene list: *ABCC9 (*NM_005691.2), *ACTA2 (*NM_001613.2), *ACTC1 (*NM_005159.4), *ACTN2 (*NM_001103.2), *AKAP9 (*NM_005751.4), *ANK2 (*NM_001148.4), *ANKRD1 (*NM_014391.2), *BAG3 (*NM_004281.3), *CACNA1C (*NM_000719.6), *CACNA2D1 (*NM_000722.2), *CACNB2 (*NM_201590.2), *CALM1 (*NM_006888.4), *CASQ2 (*NM_001232.3), *CAV3 (*NM_033337.2), *CBS (*NM_000071.2), *COL3A1 (*NM_000090.3), *COL5A1 (*NM_000093.4), *COL5A2 (*NM_000393.3), *CRYAB (*NM_001885.1), *CSRP3 (*NM_003476.3), *DES (*NM_001927.3), *DMD (*NM_004006.2), *DSC2 (*NM_024422.3), *DSG2 (*NM_001943.3), *DSP (*NM_004415.2), *EMD (*NM_000117.2), *EYA4 (*NM_004100.4), *FBN1 (*NM_000138.4), *FBN2 (*NM_001999.3), *FKTN (*NM_001079802.1), *FLNA (*NM_001456.3), *FXN (*NM_000144.4), *GATA4 (*NM_002052.3), *GATAD1 (*NM_021167.3), *GLA (*NM_000169.2), *GPD1L (*NM_015141.3), *HCN4 (*NM_005477.2), *JAG1 (*NM_000214.2), *JPH2 (*NM_020433.4), *JUP (*NM_002230.2), *KCND3 (*NM_004980.4), *KCNE1 (*NM_000219.3), *KCNE2 (*NM_172201.1), *KCNE3 (*NM_005472.4), *KCNH2 (*NM_000238.3), *KCNJ2 (*NM_000891.2), *KCNJ5 (*NM_000890.3), *KCNJ8 (*NM_004982.2), *KCNQ1 (*NM_000218.2), *LAMA4 (*NM_002290.3), *LAMP2 (*NM_002294.2), *LDB3 (*NM_007078.2), *LMNA (*NM_005572.3), *MED12 (*NM_005120.2), *MYBPC3 (*NM_000256.3), *MYH11 (*NM_002474.2), *MYH6 (*NM_002471.3), *MYH7 (*NM_000257.2), *MYL2 (*NM_000432.3), *MYL3 (*NM_000258.2), *MYLK (*NM_053025.3), *MYOZ2 (*NM_016599.4), *MYPN (*NM_032578.2), *NEXN (*NM_144573.3), *NKX2-5 (*NM_004387.3), *NOTCH1 (*NM_017617.3), *PKP2 (*NM_004572.3), *PLN (*NM_002667.3), *PLOD1 (*NM_000302.3), *PRKAG2 (*NM_016203.3), *PRKG1 (*NM_006258.3), *PTPN11 (*NM_002834.3), *RAF1 (*NM_002880.3), *RBM20 (*NM_001134363.1), *RYR2 (*NM_001035.2), *SCN1B (*NM_001037.4), *SCN2B (*NM_004588.4), *SCN3B (*NM_018400.3), *SCN4B (*NM_174934.3), *SCN5A (*NM_198056.2), *SKI (*NM_003036.3), *SLC2A10 (*NM_030777.3), *SMAD3 (*NM_005902.3), *SMAD4 (*NM_005359.5), *SNTA1 (*NM_003098.2), *TAZ (*NM_000116.3), *TBX1 (*NM_080647.1), *TBX20 (*NM_001077653.2), *TBX5 (*NM_000192.3), *TCAP (*NM_003673.3), *TGFB2 (*NM_003238.3), *TGFB3 (*NM_003239.2), *TGFBR1 (*NM_004612.2), *TGFBR2 (*NM_003242.5.), *TMEM43 (*NM_024334.2), *TMPO (*NM_003276.2), *TNNC1 (*NM_003280.2), *TNNI3 (*NM_000363.4), *TNNT2 (*NM_001001430.1), *TPM1 (*NM_001018005.1), *TRDN (*NM_006073.2), *TRPM4 (*NM_017636.3), *TTN (*NM_003319.4), *TTR (*NM_000371.3), *TXNRD2 (*NM_006440.3), *VCL (*NM_014000.2)
